# Dementia Caregivers’ Perception of Caregiver-Centered Hospice Communication: Does Experience Matter?

**DOI:** 10.1093/geroni/igaf122.2608

**Published:** 2025-12-31

**Authors:** Oonjee Oh, Karla Washington, Debra Oliver, George Demiris

**Affiliations:** University of Pennsylvania, Philadelphia, Pennsylvania, United States; Washington University in St. Louis, St. Louis, Missouri, United States; Washington University in St. Louis, St. Louis, Missouri, United States; University of Pennsylvania, Philadelphia, Pennsylvania, United States

## Abstract

While transitioning to hospice can be challenging for dementia caregivers, those new to caregiving may struggle more as they adjust to new responsibilities. Since hospice includes caregivers within the unit of care, we compared the perceptions of caregiver-centered communication with the hospice team including caregivers with different levels of experiences in caregiving and hospice. We analyzed the pre-intervention data from an ongoing cognitive-behavioral intervention targeting caregivers of hospice patients with dementia, labelled ENCODE (Empowering Caregivers of Patients with Dementia). Caregiving experience was categorized as less than 1 year, between 1 to 3 years, and over 3 years. Hospice experience was dichotomized into 4 weeks or less, and more than 4 weeks. Based on these categories, caregivers’ experiences in caregiving and hospice were grouped into 6 classes. Among 281 caregivers (mean age=59.7 (standard deviation (SD)=12.7), female 82.2%), 21.4% were new to caregiving and enrolled in hospice for 4 weeks or less. The quality of the hospice team communication was measured using the caregiver-centered communication questionnaire (CCCQ). The mean CCCQ score in our sample was 117.4 (SD = 23.1). The analysis of covariance model controlled for age and sex did not demonstrate significant differences in the CCCQ scores across the 6 classes (p value=0.07). The 5 subscales of the CCCQ (e.g., exchange of information, responding to emotions) also showed no significant differences. Such results provide an evidence base supporting the hospice model in addressing the diverse needs and concerns of dementia caregivers, regardless of the level of their prior experience with caregiving and hospice.

